# Characterization of a naturally-occurring p27 mutation predisposing to multiple endocrine tumors

**DOI:** 10.1186/1476-4598-9-116

**Published:** 2010-05-21

**Authors:** Sara Molatore, Eva Kiermaier, Christian B Jung, Misu Lee, Elke Pulz, Heinz Höfler, Michael J Atkinson, Natalia S Pellegata

**Affiliations:** 1 Institute of Pathology, Helmholtz Zentrum München, Ingolstaedter Landstrasse 1, 85764 Neuherberg, Germany; 2 Research Institute of Molecular Pathology (IMP), Dr. Bohr-Gasse 7, 1030 Vienna, Austria; 3 Medical Department - Molecular Cardiology, Klinikum rechts der Isar and Deutsches Herzzentrum - Technical University of Munich, Ismaninger Strasse 22, 81675 Munich, Germany; 4 Institute of Pathology, Technische Universität München, Trogerstrasse 18, 81675 Munich, Germany; 5 Institute of Radiation Biology, Helmholtz Zentrum München, Ingolstaedter Landstrasse 1, 85764 Neuherberg, Germany

## Abstract

**Background:**

p27Kip1 (p27) is an important negative regulator of the cell cycle and a putative tumor suppressor. The finding that a spontaneous germline frameshift mutation in *Cdkn1b *(encoding p27) causes the MENX multiple endocrine neoplasia syndrome in the rat provided the first evidence that *Cdkn1b *is a tumor susceptibility gene for endocrine tumors. Noteworthy, germline p27 mutations were also identified in human patients presenting with endocrine tumors. At present, it is not clear which features of p27 are crucial for this tissue-specific tumor predisposition in both rats and humans. It was shown that the MENX-associated *Cdkn1b *mutation causes reduced expression of the encoded protein, but the molecular mechanisms are unknown. To better understand the role of p27 in tumor predisposition and to characterize the MENX animal model at the molecular level, a prerequisite for future preclinical studies, we set out to assess the functional properties of the MENX-associated p27 mutant protein (named p27fs177) *in vitro *and *in vivo*.

**Results:**

*In vitro*, p27fs177 retains some properties of the wild-type p27 (p27wt) protein: it localizes to the nucleus; it interacts with cyclin-dependent kinases and, to lower extent, with cyclins. In contrast to p27wt, p27fs177 is highly unstable and rapidly degraded in every phase of the cell-cycle, including quiescence. It is in part degraded by Skp2-dependent proteasomal proteolysis, similarly to p27wt. Photobleaching studies showed reduced motility of p27fs177 in the nucleus compared to p27wt, suggesting that in this compartment p27fs177 is part of a multi-protein complex, likely together with the degradation machinery. Studies of primary rat newborn fibroblasts (RNF) established from normal and MENX-affected littermates confirmed the rapid degradation of p27fs177 *in vivo *which can be rescued by Bortezomib (proteasome inhibitor drug). Overexpression of the negative regulators microRNA-221/222 plays no role in regulating the amount of p27fs177 in RNFs and rat tissues.

**Conclusion:**

Our findings show that reduced p27 levels, not newly acquired properties, trigger tumor formation in rats, similarly to what has been observed in mice. The molecular characteristics of p27fs177 establish MENX as a useful preclinical model to evaluate compounds that inhibit p27 degradation for their efficacy against endocrine tumors.

## Background

The putative tumor suppressor p27Kip1 (referred to as p27) controls the progression from G1 to the S phase by regulating the activity of cyclinE/and cyclinA/Cdk2 complexes [1]. Several external signals regulate the intracellular level of p27 by either causing its increase (i.e. serum deprivation, TGFβ, contact inhibition) or its decrease (serum stimulation, estrogen, PDGF and others), thereby rendering p27 a central mediator of mitogenic and anti-mitogenic signals [2]. In addition to its negative role in cell cycle progression, p27 is involved in cell migration, neuronal differentiation and apoptosis [3-5]. Through studies of a mouse strain expressing a p27 protein impaired in cyclin/Cdk binding it has been demonstrated that p27 has a pro-oncogenic effect when it cannot bind to cyclin/Cdk complexes [6].

The intracellular level of p27 is regulated at the transcriptional, translational and post-translational level [7,8], but the best known mechanism is ubiquitin-mediated proteasomal degradation. Two main pathways involved in p27 degradation have been identified. The first is mediated by the Skp2-dependent SCF (skp-cullin-f-box) E3 ligase: phosphorylation of p27 by cyclinE/Cdk2 at a conserved threonine (Thr187) creates a binding site for Skp2, which allows polyubiquitylation and subsequent proteasomal degradation of p27. This degradation pathway is active in the nucleus of G1-S and G2 phase cells [3-5,9]. The second pathway is mediated by the KPC ubiquitin ligase and is responsible for the degradation of p27 in the cytoplasm at the G0-G1 transition [10].

Phosphorylation at specific residues regulates the activity of p27: phosphorylation at serine (Ser) 10 regulates its subcellular localization and stability [11,12]. Studies of p27S10A (serine 10 substituted by alanin) knock-in mice demonstrated that phosphorylation at Ser10 stabilizes p27 during quiescence by affecting its ability to bind to cyclin-CDK complexes [13]. Ser10 phosphorylation also triggers the export of p27 from the cell nucleus to the cytoplasm upon mitogenic stimuli, thereby allowing the protein's degradation by the KPC ubiquitin ligase [14]. Phosphorylation of p27wt at Thr187, as mentioned above, targets p27 for proteasomal-mediated degradation [15], while phosphorylation at Thr198 prevents ubiquitin-dependent degradation of free p27 and regulates the stability of p27 in G0 phase [16].

We recently identified a *Cdkn1b *germline frameshift mutation as the cause of a recessive multiple endocrine neoplasia (MEN)-like syndrome (named MENX) in the rat [17]. Rats affected by this syndrome (homozygous mutants) share phenotypic features with the p27 -/- knock-out mice (increase in size, pituitary tumors) but show additional neuroendocrine tumors (adrenals, thyroid, parathyroid). Interestingly, we and others identified *CDKN1B *germline mutations in patients with a MEN type 1 (MEN1)-like features, thereby establishing a direct link between p27 alterations and tumor predisposition also in humans (MEN4; OMIM # 610755) [17-19]. Germline *CDKN1B *mutations are a rare event in patients with an MEN1-like phenotype (estimated to be around 1.5-2.8% of cases) [18,19], and some studies failed to identify them [20,21]. So far, five germline *CDKN1B *mutations have been reported and the *in vitro *functional studies performed on three of them show that these alterations affect the ability of the encoded protein to bind its usual protein partners (p27P95S variant) or affect its amount (ATG-7G>C; stop->Q) [19]. Interestingly, the phenotype of the stop->Q germline mutation can be rescued by proteasomal inhibition *in vitro *[19].

In the MENX model, p27 protein expression is reduced or absent in the normal tissues of the mutant rats, but it becomes detectable in some advanced neoplastic lesions [17]. Therefore, this mutation is not equivalent to a null allele and it may possess specific biological functions within this model system. The mechanisms causing reduced p27 expression in the MENX-affected rats are currently unknown.

In humans, neuroendocrine tumors are uncommon neoplasms and therefore comprehensive molecular studies are difficult to perform and clinical trials are challenging to set up. In addition, there are few suitable animal models of neuroendocrine tumors and this has hampered the development and preclinical testing of novel targeted therapeutic approaches against these tumors. In this scenario, the MENX model may be of relevance to identify novel molecular mechanisms in neuroendocrine tumorigenesis and to perform preclinical therapy-response studies. An in depth knowledge of the functional consequences of the underlying genetic mutation in *Cdkn1b *is a prerequisite to further exploit the MENX animal model in preclinical studies. Moreover, since germline *CDKN1B *mutations have been found in patients, studies of the molecular phenotype of the rat mutation may broaden our understanding of the role of p27 in tumor predisposition in humans as well. Therefore, we set out to characterize the MENX-associated p27 mutant protein *in vitro *and in the patients tissues. Our studies extend to the rats the observation that the amount of functional p27 is crucial for promoting neuroendocrine tumorigenesis, event that so far had been formally proven only in genetically engineered mice.

## Results

### The MENX mutation in Cdkn1b affects p27 stability

**Figure 1:**
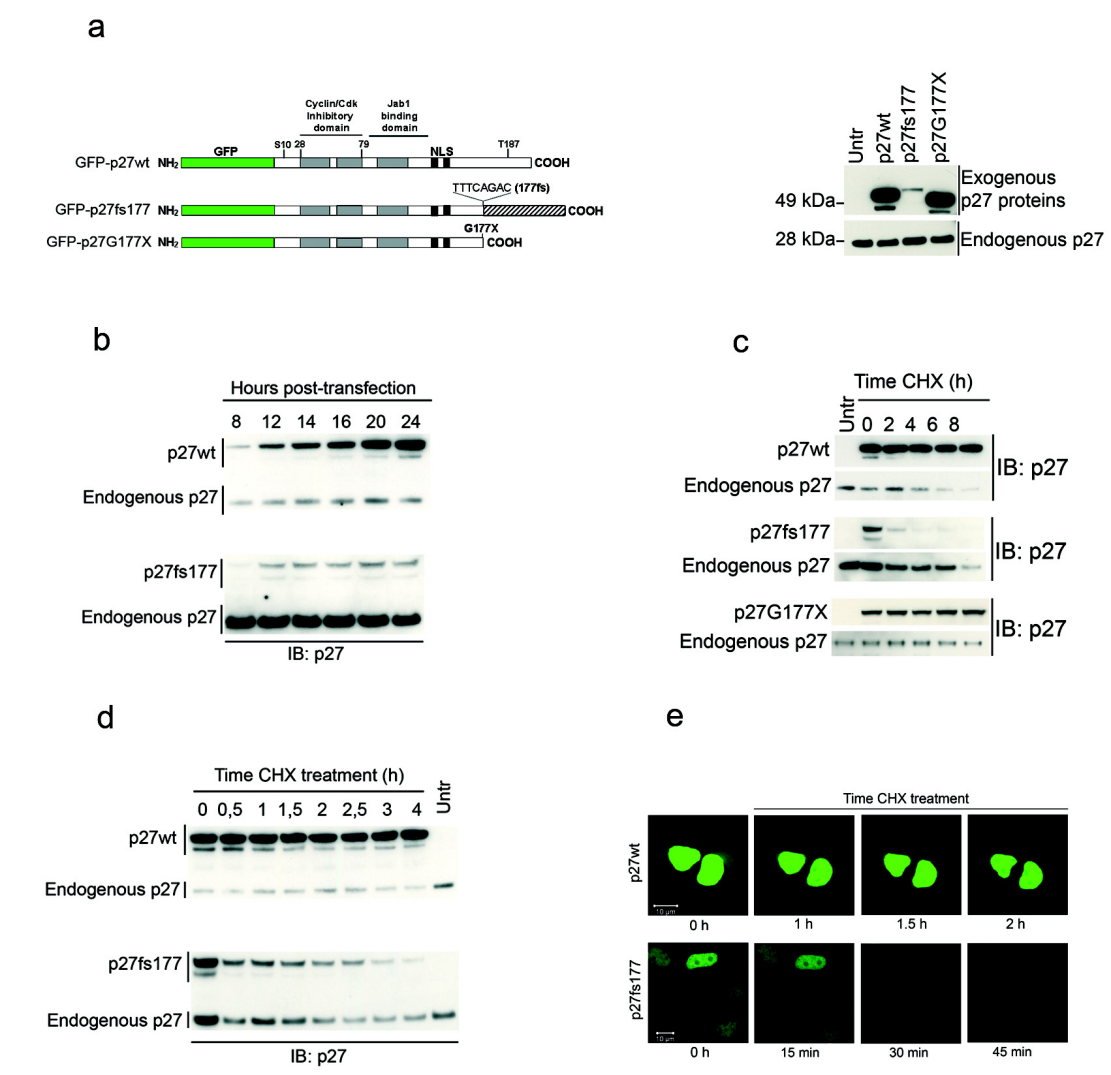
**The p27fs177 mutant protein is expressed at low level *in vitro *due to enhanced degradation**. (**a**) Left: Schematic representation of the constructs used. Right: Steady-state level of the three transfected p27 proteins: GFP-p27wt, -p27fs177 and -p27G177X. (**b**) The level of p27fs177 does not increase with time post-transfection. p27wt and p27fs177 steady-state levels were analyzed at different times after transfection into MCF7 cells by Western blotting as in A. On the left, p27wt or p27fs177 indicate the transfected proteins. (**c**) p27fs177 is unstable compared to p27wt. Kinetic of degradation of p27wt, p27fs177 and p27G177× proteins determined after transfection into asynchronously growing MCF7 cells and treatment with cycloheximide (CHX) for the indicated times. (**d**) p27fs177 has a half-life of about 1 hr. Transfected MCF7 cells were incubated with CHX for shorter time compared to panel C. (**e**) Live cell imaging of MCF7 cells transfected with p27wt or p27fs177 and treated for the indicated times with CHX confirms the rapid degradation of p27fs177. Wt, wild-type; Untr, untransfected; IB, immunoblot; Exo, exougenous; Endo, endogenous; GFP, green fluorescent protein; CHX, cycloheximide.

The MENX-associated spontaneous p27 mutation is a tandem duplication of eight nucleotides in exon 2 of *Cdkn1b *(c. 520-528dupTTTCAGAC) starting at codon 176 [17]. The mutated mRNA encodes for a predicted protein 218 amino acid long, containing a p27-unrelated C-terminal domain and referred to as p27fs177 (Figure 1a). Previously we showed that the amount of p27fs177 in normal tissues of affected rats (p27 mut/mut) is extremely low, but it may increase in areas of advanced tumors [17]. The amount of *Cdkn1b *mRNA is similar in mutant and normal rat tissues [17]. To investigate the functional properties of p27fs177, we generated vectors that express p27fs177 or wild-type p27 (p27wt) as fusion proteins with the green fluorescence protein (GFP). We also generated a protein with a premature stop codon at aminoacid 177 (named p27G177X) to evaluate the role of the p27-unrelated C-terminal domain (Figure 1a).

Upon transfection, all fusion proteins were expressed and their size was consistent with the predicted protein length (Figure 1a). p27fs177 was expressed at much lower level then either p27wt or p27G177X. A time-course experiment showed that there is no time-dependent accumulation of p27fs177 (Figure 1b). Using immunofluorescence staining, we previously showed that a Myc-tagged p27fs177 seems to localize to the nucleus upon transfection of Rat2 cells, which express endogenous p27wt, [17]. To avoid misinterpretation of the results due to possible interference of the endogenous p27 protein, we set out to assess the localization of p27fs177 in exponentially growing or serum-starved p27 knockout mouse embryonal fibroblasts (MEFs). Twenty-four hours after transfection the proteins are located mainly in the nucleus, regardless of whether the MEF cells are proliferating or growth-arrested (see Additional File 1).

We then tested whether the MENX mutation affects p27 steady-state abundance by changing its rate of degradation. The rate of p27 turnover was measured in exponentially growing MCF7 cells using cycloheximide (CHX) to block new protein synthesis. The endogenous p27 protein served as control for the treatment's efficacy. p27wt and p27G177× showed no decrease during this time course (0 h to 8 h), whereas p27fs177 was barely detectable already 2 hr after treatment (Figure 1c). The fast kinetic of degradation of p27fs177 was confirmed by shorter incubation with CHX (Figure 1d). Longer CHX treatments confirmed the higher stability of p27wt and p27G177× (half-life of ca. 10 hr) (data not shown). Live cell imaging of MCF7 cells transfected with p27wt or p27fs177, and treated with CHX 24 hr post-transfection, confirmed the very fast degradation of the mutant p27 protein (Figure 1e). Throughout this time frame (from 15 min up to 2 hr) p27fs177-associated fluorescence was detected only in the nucleus, suggesting that the protein is degraded in this compartment.

**Figure 2:**
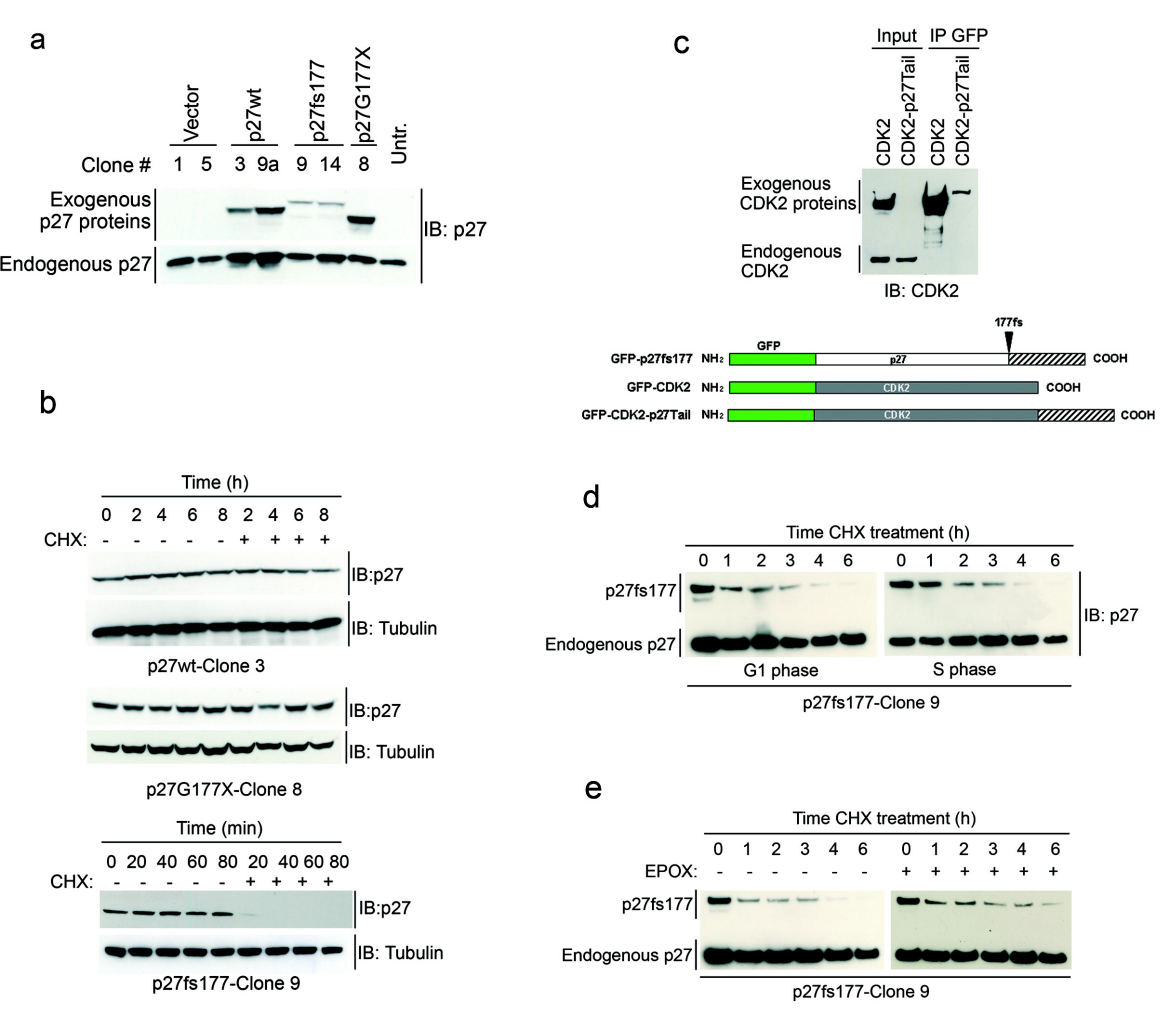
**p27fs177 is degraded very fast also in cells stably-transfected with the GFP fusion constructs**. (**a**) Clones stably expressing the various p27 mutants. (**b**) CHX treatment for clones stably expressing p27wt, p27fs177 and p27G177X. Only the exogenous p27 proteins are shown. (**c**) The p27-unrelated, C-terminal domain of p27fs177 is responsible for the rapid degradation of the protein. The CDK2 expression vectors shown on the right were transfected into MCF7 cells and protein extracts were immunoprecipitated (IP) overnight with anti-GFP antibody. (**d**) p27fs177 is very rapidly degraded independently from the cell cycle phase. p27fs177 turn-over determination in G1-phase (G1), and S-phase (S) in Clone 9 cells. (**e**) p27fs177 is in part degraded through the proteasome. Proliferating p27fs177-Clone 9 cells were treated with CHX and epoxomycin (EPOX, +) or DMSO (-) for the indicated times. Untr, untransfected; IB, immunoblot; IP, immunoprecipitation; EPOX, epoxomycin.

To study in more detail the kinetic of degradation of p27fs177, we transfected the constructs in Figure 1a into MCF7 cells and selected clones constitutively expressing the various proteins (Figure 2a). Upon incubation of these clonal cell lines with CHX, we obtained results similar to those observed in transiently-transfected MCF7 cells (Figure 2b).

The truncated p27G177× protein is as stable as p27wt, suggesting that the p27-unrelated C-terminal domain of p27fs177 must be responsible for its fast degradation. To verify this hypothesis, we cloned the tail of p27fs177, in frame, at the C-terminus of a different protein, namely CDK2, to generate the CDK2-p27tail cDNA (Figure 2c and Additional File 2). We then transfected constructs expressing the wild-type CDK2 or the CDK2-p27tail cDNAs into MCF7 cells and observed that while exogenous CDK2 is expressed at high level, the CDK2-p27tail protein cannot be detected by western blotting (Figure 2c). Following immunoprecipitation, we can detect by western blotting a faint band having a size consistent with the predicted CDK2-p27tail protein length (Figure 2c). The lower expression of the CDK2-p27tail protein is not due to a lower transfection efficiency of the DNA construct, as demonstrated by co-transfection experiments with a GFP-encoding plasmid (see Additional File 3). Thus, our results suggest that p27fs177 is degraded very rapidly due to the p27-unrelated C-terminal domain. Interestingly, this domain, when located at the C-terminus, promotes the rapid degradation of an independent protein (i.e. CDK2).

### Turnover of the p27fs177 protein is independent of the cell cycle phase

It has been shown that the stability of p27 is dependent on the cell cycle phase [13]. To verify whether this applies to p27fs177 we determined the rate of its turnover in G1-phase and S-phase Clone 9 cells stably-expressing p27fs177 (Figure 2a). As p27fs177 was undetectable upon serum deprivation (G0-phase), these results were not included. The kinetic of degradation of p27fs177 is similar in both G1 and S phases of the cell cycle (Figure 2d). Similar results were also observed for p27fs177-Clone 14 (not shown).

### Proteasome-mediated degradation of p27fs177 in vitro

p27fs177 is rapidly degraded in every phase of the cell cycle. p27wt is normally degraded through ubiquitin-dependent proteasomal degradation. To verify whether the proteasome mediates p27fs177 degradation, stably transfected p27fs177-Clone 9 cells were incubated with CHX in the presence or absence of the proteasome inhibitor Epoxomycin (EPOX). Epoxomycin partially inhibits the degradation of p27fs177, although the protein level is not completely restored back to that of untreated cells (Figure 2e). Similar results were obtained using a chemically distinct proteasome inhibitor, MG132 (not shown).

**Figure 3:**
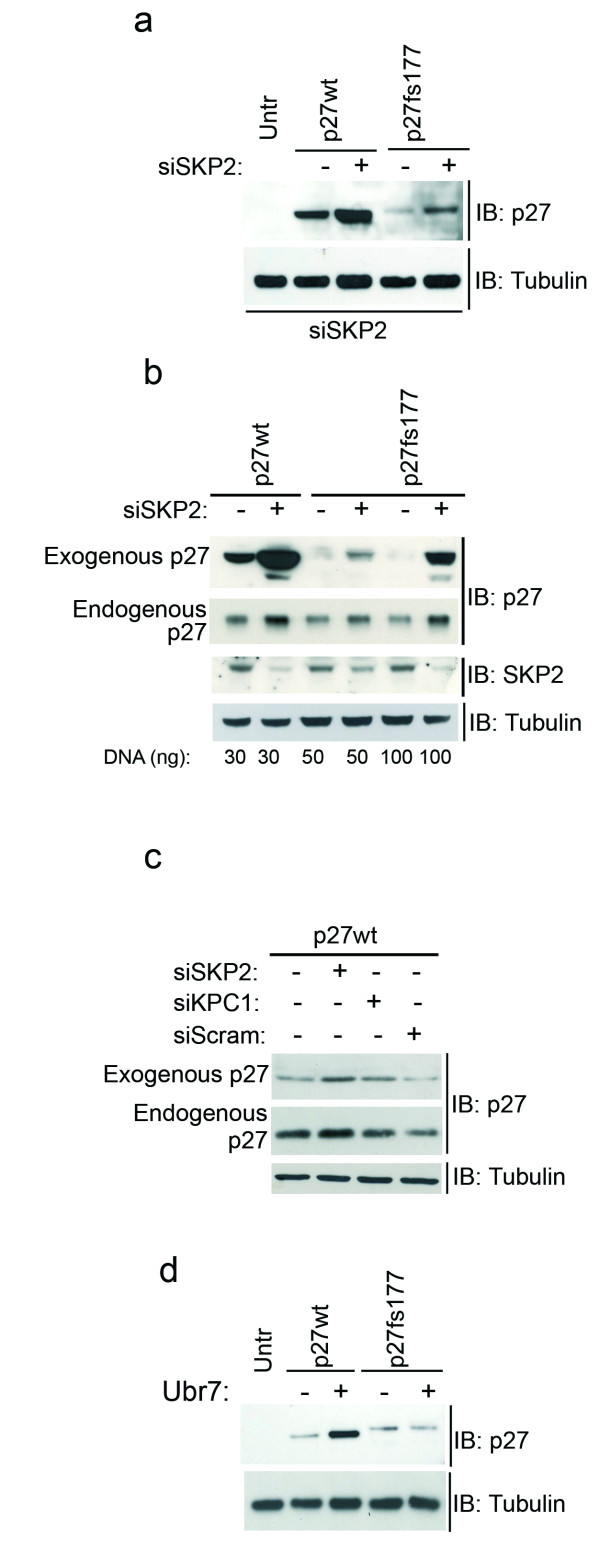
**Degradation of p27fs177 occurs through Skp2-dependent pathways**. (**a**) Expression of p27 and α-tubulin (control) were assessed in siRNA-treated and untreated cells. (**b**) Validation of efficient knockdown of Skp2 using different DNA concentrations. Probing the membrane with α-tubulin ensured equal protein loading. (**c**) To verify the specificity of the siRNA-mediated knock-down of Skp2, a scrambled siRNA oligo was transfected in parallel with p27wt plasmid. siRNA for knock-down of *KPC1 *was included as positive control as this protein also degrades p27wt. Probing the membrane with α-tubulin ensured equal protein loading. (**d**) A ubiquitin mutant (pUbr7) co-transfected in parallel with the p27wt and p27fs177 expression vectors has no effect on p27fs177 degradation.

p27fs177 is unstable due to its C-terminal domain and is degraded through the proteasome. This could occur because this protein is recognized as misfolded by the proteasome or could occur through the canonical ubiquitin-dependent pathways that mediate the degradation of wild-type p27. This is an important issue to clarify if we want to exploit this animal model to evaluate *in vivo *compounds aimed at restoring p27 function in the tumor cells by interfering with its degradation. As mentioned above, p27 is degraded in the nucleus by Skp2_SCF E3-mediated ubiquitination followed by proteasomal degradation. To determine whether Skp2 is involved in the turnover of p27fs177, we transfected the above constructs into exponentially growing MCF7 cells and reduced Skp2 expression by siRNA-mediated knock-down (Figure 3a). Reduction of Skp2, as confirmed by western blotting (Figure 3b), led to an increase in the levels of both p27wt and p27fs177, whereas transfection of scrambled siRNA did not increase p27wt levels, attesting to the specificity of the Skp2-siRNA (Figure 3c). Similar results were obtained using a pool of different siRNA oligos against Skp2 (Additional File 4). Therefore, p27fs177 is still a substrate of ubiquitination by the Skp2-SCF complex despite the absence of the Thr187 residue. This is an interesting observation, as it has been amply reported that phosphorylation at Thr187 creates the recognition site for Skp2 binding.

If polyubiquitination is a pre-requisite for proteosomal turnover, then inhibition of the ubiquitination pathway should stabilize p27fs177. We tested this hypothesis using a ubiquitin mutant (UbR7) that inhibits ubiquitin chain elongation, allowing only monoubiquitination [22]. Cotransfection of this mutant ubiquitin increased the steady-state abundance of p27wt (control) but not that of p27fs177 (Figure 3d). This data suggests that polyubiquitination is required for p27wt but not for p27fs177 degradation, although we cannot rule out the possibility that these proteins are differentially sensitive to conjugation with UbR7.

In summary, the degradation of p27fs177 is, at least in part, mediated by the ubiquitin ligase Skp2. However, the relative differences between p27wt and p27fs177 protein levels remained under conditions of Skp2 knock-down, indicating that p27fs177 is degraded also by Skp2-independent mechanisms. We speculate that p27fs177 is directly degraded by the proteasome due to its altered conformation.

### The p27fs177 protein is not phosphorylated on Ser10 but retains the ability to interact with Cdk2 and Cdk4

Studies of p27S10A knock-in mice (where Ser10 is substituted by alanine) demonstrated that phosphorylation of p27 on Ser10 normally stabilizes the protein in quiescence by decreasing its ability to bind to cyclin-CDK complexes [13]. As p27fs177 is very unstable in G0, we asked whether this was due to lack of phosphorylation on Ser10. Hela cells were transfected with the p27wt and p27fs177, and with the control p27S10A mutant that cannot be phosphorylated at this residue. While p27wt can be detected using the anti-phospho-Ser10 antibody, both p27S10A and p27fs177 cannot be (see Additional File 5a). As p27fs177 is expressed at low level upon transfection, we also performed immunoprecipitation with the anti-phospho-Ser10 antibody to increase the sensitivity of the method. We observed that only p27wt can bind the phospho-specific antibody (see Additional File 5a). Therefore, p27fs177 is not phosphorylated at Ser10 or the proportion that is phosphorylated is below detection. Thus, lack of (or extremely reduced) phosphorylation at Ser10 may concur in making the p27fs177 protein unstable.

It has been shown that the formation of heterotrimeric p27-cyclin-Cdk complexes is required for p27 degradation as mutations that abolish the association with cyclin-Cdks stabilize p27 [13]. Thus, we next decided to determine whether p27fs177 retains the ability to interact with Cyclin-Cdk complexes. Serum-starved and exponentially growing p27fs177-Clone 9 cells were used for co-immunoprecipitation (IP) studies. The binding of p27fs177 to Cyclin D1, Cyclin E, Cdk2 and Cdk4 was determined by western blotting. The results show that p27fs177 retains the ability to interact with all proteins albeit with different efficiencies (see Additional File 5b). Due to the low level of p27fs177 present in the cells, the amount of the Cdks bound to is accordingly low. p27fs177 binds more efficiently to Cdk4 then to Cdk2.

### Nuclear dynamic of p27fs177 revealed by photobleaching

As the p27fs177 protein is degraded very fast in every phase of the cell cycle and it is detected only in the nucleus, we postulated that p27fs177 in this compartment binds to the degradation machinery. Fluorescence Recovery After Photobleaching (FRAP) is an optical technique capable of measuring the two dimensional lateral diffusion of a fluorescent molecule over time in single cells and which has been applied to study cell membrane diffusion and protein binding [23]. We used FRAP to study the diffusion coefficient of the different GFP-p27 proteins. p27wt showed in the nucleus a diffusion coefficient (D-value) of 8.8 μm^2^/sec, while p27G177× showed a D-value of 10.1 μm^2^/sec (*P *= 0.22), whereas p27fs177 had a much lower diffusion coefficient (3.78 μm^2^/sec) (*P *= 0.0012) (Table 1 and Additional File 6). Such difference in diffusion behavior was not caused by increased molecular mass of p27fs177 compared to p27wt (ca. 2.3 kDa) as FRAP recovery rates due to diffusion are weakly dependent on protein mass [23].

**Table 1:** Diffusion coefficient of the various GFP-p27 fusion proteins in the nucleus.

**Construct**	**Nuclear Diffusion**	
	
	**D (mm2/sec)**	**sd (yEr ±)**
		
GFP-p27wt	8.81	2.01
GFP-p27fs177	**3.78**	**0.15**
GFP-p27G177X	10.1	0.92

Starting at 24 hr after transfection of p27fs177 in the various recipient cell lines used, the protein is localized in the nucleus (Additional File 1). At 18-20 hr a percentage of cells (ca. 30%) still show some cytoplasmic p27fs177 that has not yet been imported into the nucleus. To determine whether also in this compartment p27fs177 shows reduced molecular dynamics, we performed FRAP to measure the diffusion coefficient of the various p27 proteins in the cytoplasm at 18 hr post-transfection of Rat2 cells. The results show that in this compartment the motility of p27wt, p27fs177 and p27G177× is similar, indicating that p27fs177 is not associated to a protein complex in the cytoplasm (Additional File 6).

### Instability of p27fs177 in primary rat cells

**Figure 4:**
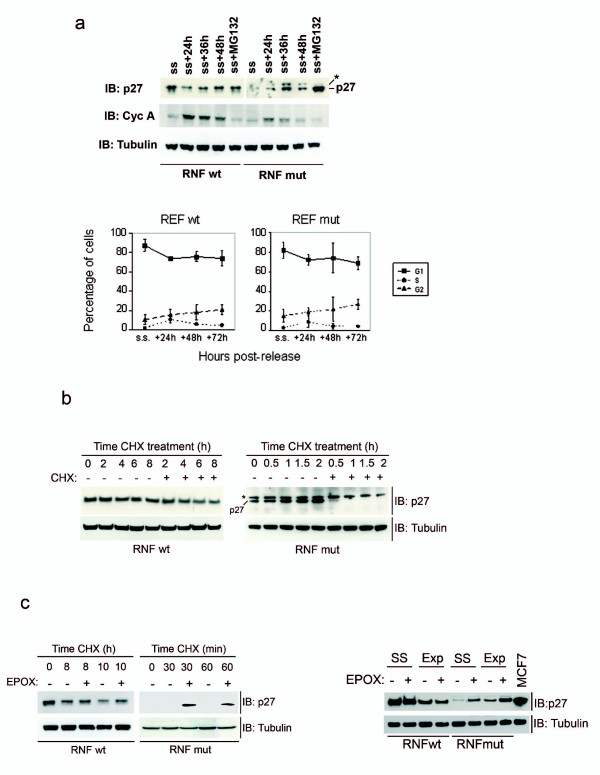
**p27fs177 is highly unstable also in primary rat fibroblasts**. (**a**) RNF mut cells progress through the cell cycle as RNF wt cells. FACS analysis of DNA content. (**b**) CHX treatment on exponentially growing RNFs. The asterisk indicates a non-specific band. (**c**) Endogenous p27fs177 is in part degraded by the proteasome. RNFs were treated with CHX and epoxomycin (+) or DMSO (-) for the indicated times. Left: Endogenous p27fs177 is so unstable that often can be detected only in the presence of proteasome inhibitors such as EPOX. Right: p27fs177 is actively degraded not only in exponentially growing (Exp) RNF mut cells but also in serum-starved cells (SS) and is stabilized by treatment with EPOX.

We could not detect ectopic p27fs177 in quiescent cells. To study the degradation of p27fs177 during quiescence in a more physiological system, we established primary skin fibroblast cell lines from newborn wild-type (RNFwt) and mutant (RNFmut) rat littermates. Serum-starvation (ss) and release experiments showed that wild-type p27 levels fluctuate throughout the cell cycle, as one might expect: they were elevated in serum-starved cells, decreased during G1 (up to 24 h), remained lower in S and G2 phases (up to 48 h) (Figure 4a). The level of p27fs177 also oscillated throughout the cell cycle but the protein could not be detected in serum-starved cells (Figure 4a). Analysis of cyclin A expression confirmed the progression of RNFs (both wt and mut) through the cell cycle (Figure 4a). The level of p27fs177 in quiescent RNFmut cells increased by adding MG132, indicating that proteasomal degradation is active also during this phase (Figure 4a).

In proliferating RNFs, p27fs177 is degraded within minutes following treatment with CHX, and if we add Epoxomycin (EPOX) p27fs177 is stabilized (Figure 4b and 4c). Therefore, the low level of endogenous p27fs177 protein in RNFs is caused by its rapid degradation mediated, at least in part, by the proteasome, as seen *in vitro*. The mutant p27 protein is even less stable in this more physiological system than *in vitro*.

In REFmut cells a band migrating slower then p27fs177 can be observed at long exposure times but since it is not affected by any treatment we performed (Figure 4c) nor is the product of abnormally-spliced *Cdkn1b *transcripts (data not shown) we consider it p27-unrelated.

**Figure 5:**
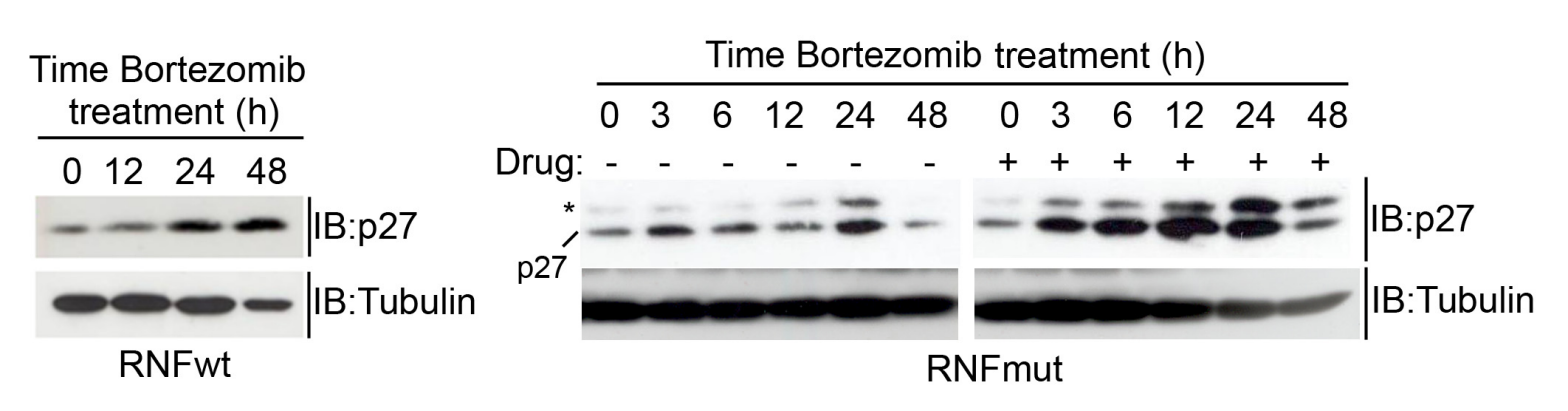
**p27fs177 can be stabilized by Bortezomib in primary rat fibroblasts**. Exponentially growing RNF wt and RNF mut cells were treated with the proteasome inhibitor Bortezomib for the indicated time. The asterisk indicates a non-specific band.

In view of possible preclinical therapy-response studies of MENX, we tested the effect of Bortezomib (Velcade^®^), a proteasome inhibitor drug currently used to treat patients with multiple myeloma and other neoplasias [24], on the stabilization of p27fs177. We detected an increase in the level of p27fs177 using a dose of the drug reported to elicit a biochemical response (10 nM). The restoration of p27fs177 expression lasted until 48 hr post-treatment and was evident already at 3 hr, in keeping with the usual short half-life of this protein. In contrast, p27wt, which is more stable, increases 24 hr after incubation with Bortezomib and 48 hr later the wild-type protein is still present at high level (Figure 5).

In many cell types there is an inverse correlation between p27 level and cell proliferation. To determine whether the low expression level of p27fs177 has an effect on cell cycle progression, RNFs were growth-arrested and monitored following serum stimulation over a 72-h period for cell cycle progression by flow cytometry. No significant difference in cell cycle distribution was observed by FACS analyses between RNFwt and RNFmut (Figure 4a). Thus, lack of functional p27 within the MENX model does not grossly affect cell cycle progression. This is in agreement with what has been observed in the normal tissues of MENX rats following immunohistochemical staining with the proliferation antigen Ki67 (Pellegata NS, unpublished observation).

The reduced level of p27fs177 in RNFmut is only in part explained by proteasome-mediated degradation, as it is slightly lower than that of p27wt in RNFwt cells also in presence of Epoxomycin. Recently, it has been reported that miRNA-221&222 down-regulate p27 at the post-transcriptional level in cell lines and in primary tumors [8,25]. Therefore, we checked whether the low amount of p27fs177 in MENX-affected rat tissues and cells might also be due to an up-regulation of miRNA-221&222. Analysis of the expression of miRNA-221&222 in RNFwt and RNFmut cells, as well as in a series of normal and tumor rat adrenal tissues (organ affected by the MENX syndrome), showed no significant difference in the expression of these two miRNAs between samples expressing wild-type or mutant *Cdkn1b *(see Additional File 7). Therefore, a higher expression of miRNA-221&222 does not play a role in the low level of expression of p27fs177 in either rat primary cells or rat tissues.

## Discussion

We show here that the mutant p27 protein associated with MENX is very unstable due to the p27-unrelated C-terminal domain. Its rapid degradation is, at least in part, mediated by the Skp2-dependent ubiquitination pathway, just as p27wt. Interestingly, it has been reported that Skp2-dependent ubiquitination and subsequent degradation of p27 requires the phosphorylation of Thr187, which is then recognized and bound by the Skp2 ubiquitin ligase. Since p27fs177 lacks the Thr187 residue but its degradation is still regulated by Skp2 (as seen in siRNA knock-down experiments), we have to postulate that a Skp2-dependent but Thr187-independent mechanism for p27 degradation exists, as it had been previously hypothesized based on studies of cells (fibroblasts, thymocytes) from p27T187A (Thr substituted by Ala) knock-in mice [26]. A mutant ubiquitin that inhibits ubiquitin chain elongation has no effect on the degradation of p27fs177, while it rescues wild-type p27 degradation, indicating that p27fs177 is also degraded by mechanisms different from those modulating the level of p27wt. However, what is relevant is that the expression of p27fs177 can be recovered by proteasome-inhibition. This finding, combined with the fact that p27fs177 retains the capacity to interact with cyclins and Cdks, suggests that if the expression of this protein can be rescued inhibiting the proteasome, it might resume its anti-proliferative activity.

The fact that p27fs177 retains the potential to bind to the usual partners of p27 is highly relevant: if this protein can be re-expressed in the tumor cells, it might then resume its inhibitory effect on the cell cycle and therefore prevent or reverse tumor growth *in vivo*.

Our observation that in some areas of very advanced tumors in MENX mutant rats p27fs177 is expressed at a detectable level might not be in contradiction to this hypothesis. In fact, re-expression of the protein is observed in rats whose general health status is irreversibly compromised by the detrimental effects related to tumor progression.

The slower nuclear dynamic of p27fs177 is therefore due to its being part of a multi-protein complex, likely together with the degradation machinery. This is in agreement with the finding that the nuclear Skp2 ubiquitin ligase plays a role in p27fs177 degradation.

p27 is frequently down-regulated in human tumors and in some of them increased proteasomal degradation has been identified as the cause of the reduced p27 level [27,28]. Because of these findings, efforts have been made to identify compounds that interfere with the protein turnover machinery in order to achieve p27 re-expression in cancer cells. An example is the novel compound, Argyrin A, which specifically prevents p27 degradation and holds great promise as anti-cancer drug [29]. Due to the characteristics of p27fs177 here described, MENX rats represent a useful pre-clinical model in which to test the efficacy of targeted therapeutic approaches aiming at inhibiting p27 degradation. Interestingly, an established anticancer proteasome inhibitor compound, Bortezomib, can rescue p27fs177 expression in fibroblasts of MENX-affected rats. Therefore, MENX might be also employed to test *in vivo *the efficacy of Bortezomib against neuroendocrine tumors and to study whether the potential effect of the drug is mediated by reexpression of p27 function. A Phase II clinical trial aiming at evaluating the effect of the proteasome inhibitor Bortezomib on metastatic neuroendocrine tumors failed to show any objective tumor response in this patient cohort [30]. However, the patients had not been stratified for p27 expression, measurement of the increase in p27 expression following treatment was not among the biological end points of the study and Bortezomib induces a wide range of off-target effects [27]. Thus, the efficacy of p27 re-expression in the treatement of neuroendocrine tumors is still an open question that can be addressed by studying MENX-affected rats.

## Conclusions

Our findings suggest that the p27fs177 mutant protein does not acquire novel molecular properties, and triggers tumor formation in the spontaneous rat MENX model because it behaves as a hypoactive allele. So far, this had only been formally proven in various p27 knock-out and knock-in mouse models [31-33].

The identification of the molecular phenotype of the few naturally-occurring p27 mutations so far identified in MENX and in human patients is important to better understand the link between p27 and neuroendocrine tumor predisposition (and tumorigenesis in general), and may provide clues for a targeted management of the families carrying such mutations.

## Methods

### Plasmid constructs and antibodies

The rat wild-type Cdkn1b cDNA was cloned in the pEGFP-C3 vector (BD Biosciences, Erembodegem, Belgium) in frame with the green fluorescent protein (GFP) tag. A rat *Cdkn1b *cDNA containing the MENX rat mutation (p27fs177) and a rat *Cdkn1b *cDNA containing a stop codon at position 177 (p27G177X) were generated by PCR.

The rat wild-type Cdkn1b cDNA was cloned in the pEGFP-C3 vector (BD Biosciences, Erembodegem, Belgium) in frame with the green fluorescent protein (GFP) tag. A rat *Cdkn1b *cDNA containing the MENX rat mutation (p27fs177) and a rat *Cdkn1b *cDNA containing a stop codon at position 177 (p27G177X) were generated by PCR.

The pUbr7 construct was gently provided by N. Malek

Primary antibodies used were: anti-p27 monoclonal antibody (BD Biosciences); anti-Skp2 monoclonal antibody; anti-Cyclin A polyconal antibody (Santa Cruz Biotechnology, Santa Cruz, CA, USA). To control for equal protein loading, α-tubulin immunostaining (Santa Cruz) was performed.

### Cell culture, transfections, protein extraction and Western blotting

MCF7 (human breast cancer) cell line was maintained in RPMI 1640 medium supplemented with 10% fetal bovine serum, 20 mM L-glutamine, 100 units/ml of penicillin G sodium, and 100 μg/ml streptomycin. When establishing stable transfectants, cells were selected by adding 0.4 mg/ml G418 to the culture medium.

Primary rat fibroblast cells were obtained from the epidermis of newborn rats having a p27 wt/wt (RNF wt) or a p27fs177/p27fs177 genotype (RNF mut) and were maintained in DMEM medium supplemented with 10% fetal bovine serum, 20 mM L-glutamine, 100 units/ml of penicillin G sodium, 100 μg/ml streptomycin, and Fungizone^® ^antimycotic (Invitrogen, Darmstadt, Germany).

The cells were grown in a humidified atmosphere containing 5% CO_2 _at 37°C.Cells were growth arrested by decreasing the serum concentration in the medium to 0.1% for 72 h and then released by serum addition,

Asynchronously growing cells were transiently transfected with the different constructs when 70-80% confluent using the FuGene HD reagent (Roche Applied Bioscience, Basel, Switzerland). Cells were collected and lysed 24 h later in protein lysis buffer (10 mmol/l Tris-HCl pH 7·4, 5 mmol/l EDTA, 130 mmol/l NaCl, 1% Triton, and 1× Mini-Complete protease inhibitors cocktail, Roche).

Equal protein amounts (50 μg) were resolved on 4-15% SDS-PAGE pre-cast gels (Invitrogen, Darmstadt, Germany).

### Drug treatments and RNA interference

The cells were treated with 25 μg/mL cycloheximide (Sigma-Aldrich Biochemie, Hamburg, Germany), 10 μM epoxomycin (Enzo Life Sciences, Lörrach, Germany), or 10 nM Bortezomib (LC Labs, Woburn, MA, USA).

For RNAi studies, short interfering RNA (siRNA) duplexes specific for *Skp2 *(n = 4) (1 μg; siGenome SMARTpool, Dharmacon, Lafayette, CO, USA) and *KPC1 *(1 μg each; ID 133486, 133487 Ambion-Applied Biosystems, Darmstadt, Germany) were obtained and transfected into MCF7 cells using X-tremeGENE reagent (Roche) together with expression plasmids for p27wt (30 ng) or p27fs177 (50 ng). To verify the specificity of the siRNA-mediated knock-down, a scrambled siRNA oligo was transfected in parallel.

### Immunofluorescence

MCF7 cells were plated on dishes having a cover slip for microscopy (MatTek Corporation, Ashland, MA, USA) and transfected as above with p27fs177 and p27wt protein constructs. To re-create a more physiological condition (37°C and 5% CO2), dishes were put under a micro-incubation chamber (Zeiss, Jena, Germany) mounted on a confocal laser scanning microscope (Zeiss). Images were taken every 15 min using always the same settings.

### Fluorescence activated cell sorting (FACS)

Cells were collected and fixed in 70% ice-cold ethanol and stored at -20°C until ready to proceed. Cells were rinsed and resuspended in 1× PBS containing 5 μg/ml propidium iodide and 150 μg/ml RNaseA. After incubation for 30 min at 37°C cells were analyzed in a FACS machine (Beckman Coulter, Krefeld, Germany) for DNA content.

### Immunoprecipitations

500 μg of total protein extracted from the cells were incubated with a polyclonal anti-GFP antibody (BD Biosciences) in IP buffer (5 mM EDTA, 0.5% Triton X-100 in PBS 1×) and 20 μl of agarose resin overnight at 4°C. Unbound protein fraction was removed, the resin was washed five times with IP buffer and immunoprecipitated proteins were eluted from the agarose resin using Laemmli buffer and analyzed by Western blot.

Additional experimental procedures and associated reference are available in Additional File 8.

## Competing interests

The authors declare that they have no competing interests.

## Authors' contributions

SM carried out the molecular studies and drafted the manuscript. EK, CBJ, ML and EP carried out the molecular studies. HH and MJA participated in the design of the study. NSP conceived the study, and participated in its design and coordination and drafted the manuscript. All authors read and approved the final manuscript.

## Supplementary Material

Additional file 1 **Intracellular localization of the p27 fusion proteins**. p27 -/- mouse embryonic fibroblasts (MEF) exponentially growing (exp) or after 72 hrs of serum deprivation (s.s.) were transfected with GFP -p27wt, -p27fs177 and -p27G177X. Cells were investigated for p27 immunofluorescence 24 h after transfection. Cell nuclei were counterstained with 1 μg/ml Hoechst before mounting on slides. Click here for file

Additional file 2 **Sequence of the proteins encoded by the constructs used for the transient transfections **(see Figure 2C). The DNA sequence of the *CDK2 *human gene (NM_001798) was cloned into the pEGFP vector in frame with the *EGFP *gene. By *in vitro *mutagenesis we changed the stop codon of *CDK2 *from TGA->GGA and then we cloned in frame the 129 bp of the p27Fs177 tail, starting at the site of the insertion, in frame with the *CDK2 *cDNA. All constructs were confirmed by sequencing. Click here for file

Additional file 3 **The low expression of CDK2-p27Tail is not due to lower transfection efficiency**. MCF7 cells were co-transfected with the fusion constructs encoding GFP-CDK2, -CDK2-p27Tail, -p27WT or -p27fs177 and with an empty pEGFP vector to monitor the efficiency of transfection. Proteins were resolved and blotted with a monoclonal anti-GFP antibody. As mentioned in the article text, the CDK2-p27Tail protein is expressed at such a low level that it can be detected only following immunoprecipitation. Click here for file

Additional file 4 **Degradation of p27fs177 occurs through Skp2-dependent pathways**. For RNAi studies, short interfering RNA (siRNA) duplexes specific for *Skp2 *(n = 4) (1 mg) were transfected into MCF7 cells using X-tremeGENE reagent together with expression plasmids for p27wt (30 ng) or p27fs177 (50 ng). Immunoblotting was performed with the indicated antibodies against p27, SKP2 and α-Tubulin (to control for equal leading). Click here for file

Additional file 5 (**a**) **p27fs177 is not phosphorylated at Ser10 *in vitro***. Hela cells were transfected with the p27wt, p27fs177 and p27S10A contructs. Cells were collected 24 h later. Proteins were resolved on 4-15% SDS-PAGE pre-cast gels and blotted with the antibodies indicated on the left side: polyclonal anti-P-S10 antibody and monoclonal antibodies against p27 and against α-tubulin. The arrow indicates the p27fs177 protein. For immunoprecipitation, 500 μg of protein lysates were incubated o.n. at 4°C with 10 ml of anti-P-S10 antibody and then immunoblotted with the monoclonal anti-p27 antibody. (**b**) **p27fs177 interacts with Cdks**. Stably-transfected p27fs177-Clone 9 cells were harvested while exponentially growing (pro) or after incubation for 72 hrs in medium supplemented with 0,1% FBS (serum starved, ss) and proteins immunoprecipitated o.n. at 4°C with 10 ml of anti-GFP polyclonal antibody, 20 ml of anti-CyclinD1 polyclonal antibody or 20 ml of anti-CyclinE polyclonal antibody. Proteins were resolved as in (a) and blotted with the antibodies indicated on the right side (Cdk2 and CyclinE monoclonal, Cdk4 and CyclinD1 polyclonal). Click here for file

Additional file 6 **Photobleaching shows that p27fs177 has reduced motility in the nucleus, but not in the cytoplasm**. Diffusion coefficient of the various GFP-p27 fusion proteins in the nucleus. The appropriate diffusion values are calculated as mean values of single measurements ± standard deviation. The number of bleached cells per construct is indicated in the diagram bar (n). The number of cells analyzed for cytoplasmic diffusion is smaller than for nuclear diffusione because only a percentage of cells (ca. 30%) shows cytopalsmic localization of these proteins. The asterisk (*) indicates a significant decrease of diffusion for thep27fs177 protein compared to p27wt (P = 0.0012). FRAP, fluorescence recovery after photobleaching. Click here for file

Additional file 7 **Expression of miRNA-221& 222 in rat adrenal tissues and in primary fibroblasts (REF cells) show no difference between normal and mutated rats**. (**a**) Total RNA was extracted from normal rat adrenal tissue (wt/wt) and rat adrenal tumors (mut/mut) using Trizol (Invitrogen). Quantitation of mature miRNA-221 and 222 expression levels in and was performed by RT-PCR using TaqMan MicroRNA Assays. All RT-PCR were performed in triplicate. One endogenous control was used for the normalization of RNA input: small nucleolar RNA RNU44. The data are presented as the fold change of miRNA expression in tissues after normalization to an endogenous control (RNU44). (**b**) Exponentially growing REF7 (wt/wt) or REF10 (mut/mut) fibroblasts were collected. Total RNA extracted and TaqMan assays were performed as (a). The differences in expression between mutant and normal tissues or cells are not statistically significant. •Microsoft Power Point Presentation Click here for file

Additional file 8 **Supplementary Materials and Methods, Reference**. Microsoft Word Document Click here for file
