# Supplementary material for: Characterization of a naturally-occurring p27 mutation predisposing to multiple endocrine tumors
Source: Mol Cancer. 2010 May 21;9:116. doi: 10.1186/1476-4598-9-116 (PMC2881881; doi:10.1186/1476-4598-9-116)
Supplement: Additional file 1 — Intracellular localization of the p27 fusion proteins. p27 -/- mouse embryonic fibroblasts (MEF) exponentially growing (exp) or after 72 hrs of serum deprivation (s.s.) were transfected with GFP -p27wt, -p27fs177 and -p27G177X. Cells were investigated for p27 immunofluorescence 24 h after transfection. Cell nuclei were counterstained with 1 μg/ml Hoechst before mounting on slides. [file 1476-4598-9-116-S1.PPT]

## Slide 1
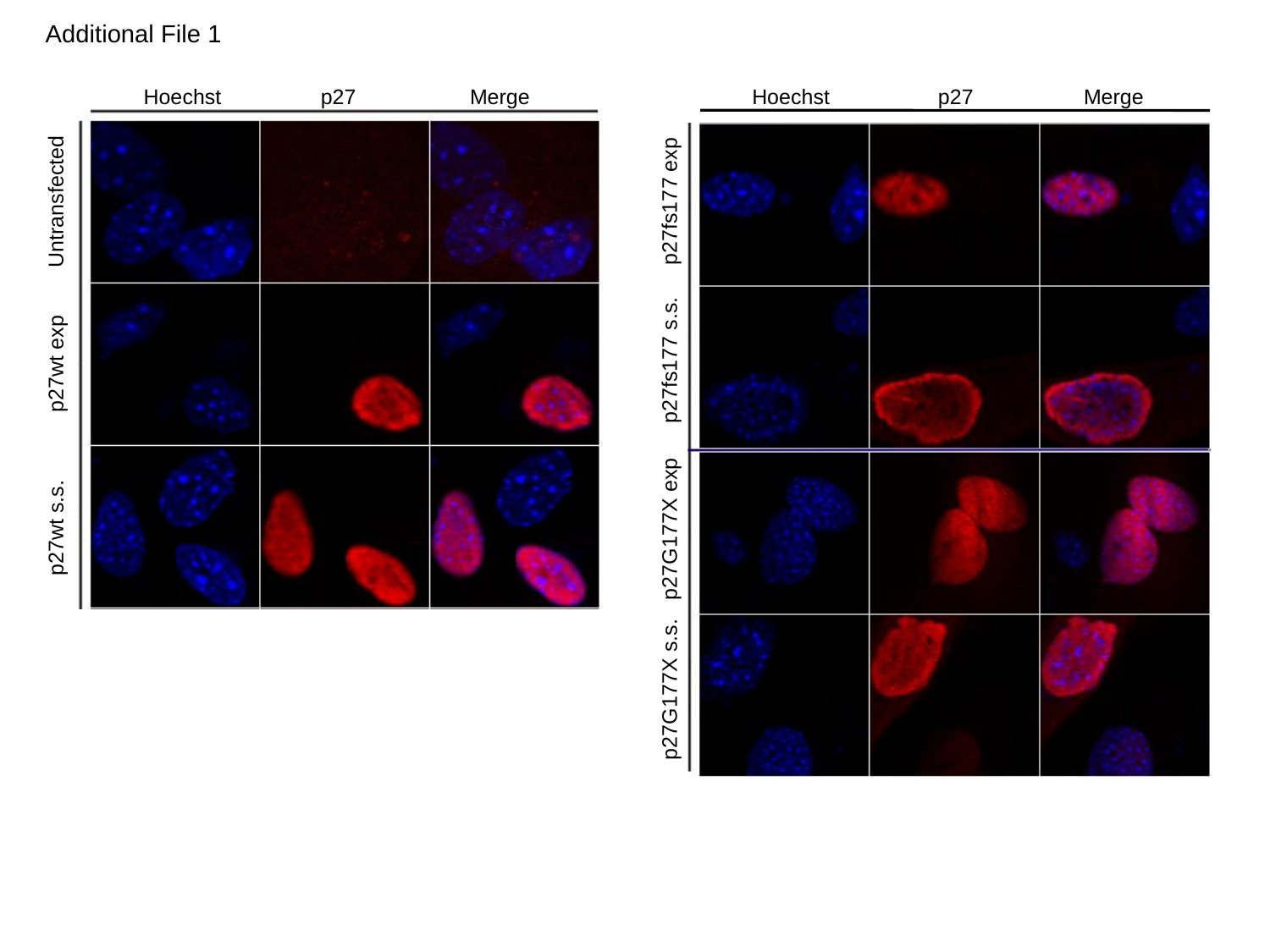

Additional File 1
Hoechst
p27
Merge
Untransfected
p27wt exp
p27wt s.s.
Hoechst
p27
Merge
p27fs177 exp
p27fs177 s.s.
p27G177X exp
p27G177X s.s.
