# Supplementary material for: Characterization of a naturally-occurring p27 mutation predisposing to multiple endocrine tumors
Source: Mol Cancer. 2010 May 21;9:116. doi: 10.1186/1476-4598-9-116 (PMC2881881; doi:10.1186/1476-4598-9-116)
Supplement: Additional file 2 — Sequence of the proteins encoded by the constructs used for the transient transfections (see Figure 2C). The DNA sequence of the CDK2 human gene (NM_001798) was cloned into the pEGFP vector in frame with the EGFP gene. By in vitro mutagenesis we changed the stop codon of CDK2 from TGA->GGA and then we cloned in frame the 129 bp of the p27Fs177 tail, starting at the site of the insertion, in frame with the CDK2 cDNA. All constructs were confirmed by sequencing. [file 1476-4598-9-116-S2.PPT]

## Slide 1
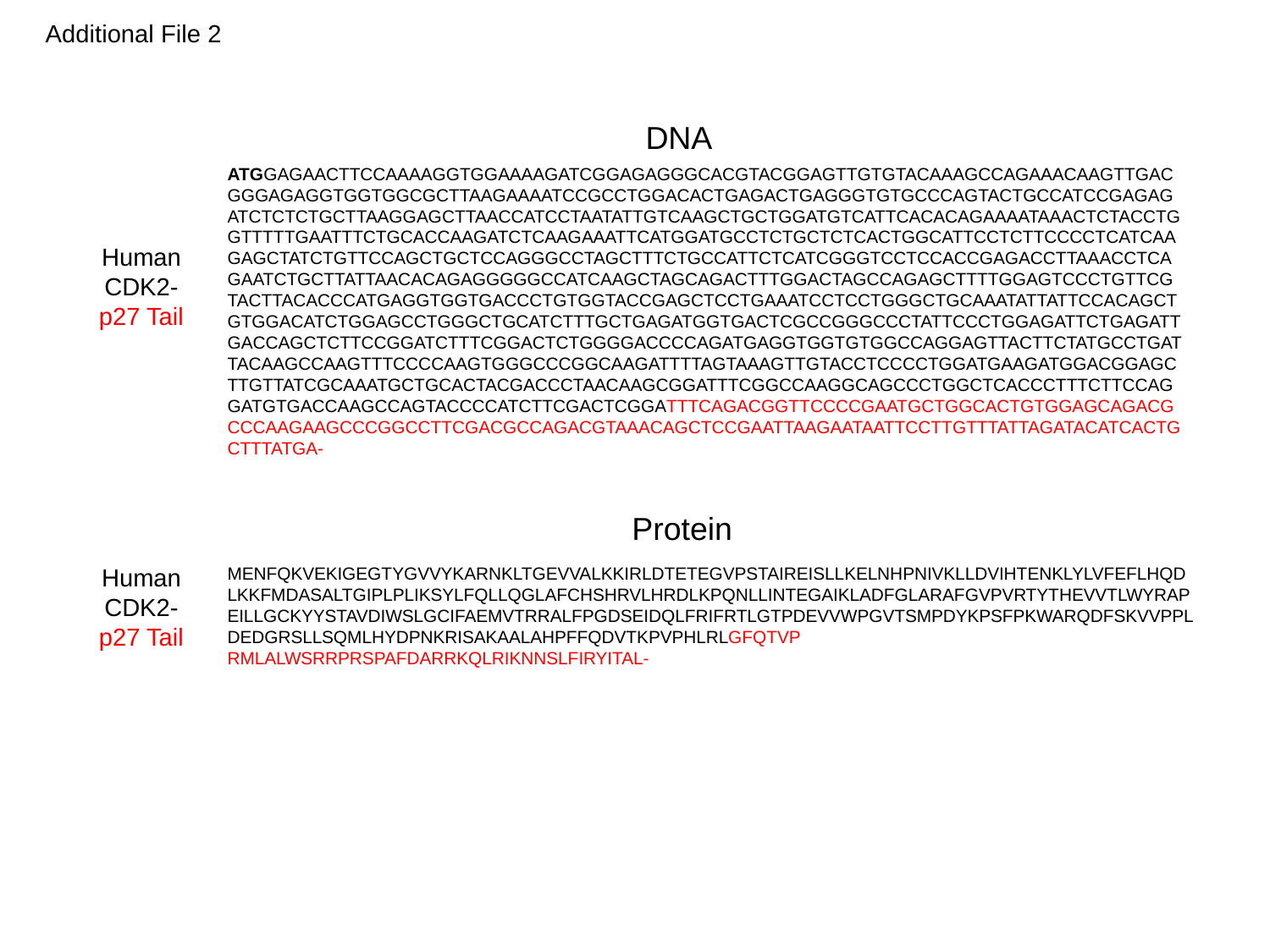

Additional File 2
DNA
ATGGAGAACTTCCAAAAGGTGGAAAAGATCGGAGAGGGCACGTACGGAGTTGTGTACAAAGCCAGAAACAAGTTGACGGGAGAGGTGGTGGCGCTTAAGAAAATCCGCCTGGACACTGAGACTGAGGGTGTGCCCAGTACTGCCATCCGAGAGATCTCTCTGCTTAAGGAGCTTAACCATCCTAATATTGTCAAGCTGCTGGATGTCATTCACACAGAAAATAAACTCTACCTGGTTTTTGAATTTCTGCACCAAGATCTCAAGAAATTCATGGATGCCTCTGCTCTCACTGGCATTCCTCTTCCCCTCATCAAGAGCTATCTGTTCCAGCTGCTCCAGGGCCTAGCTTTCTGCCATTCTCATCGGGTCCTCCACCGAGACCTTAAACCTCAGAATCTGCTTATTAACACAGAGGGGGCCATCAAGCTAGCAGACTTTGGACTAGCCAGAGCTTTTGGAGTCCCTGTTCGTACTTACACCCATGAGGTGGTGACCCTGTGGTACCGAGCTCCTGAAATCCTCCTGGGCTGCAAATATTATTCCACAGCTGTGGACATCTGGAGCCTGGGCTGCATCTTTGCTGAGATGGTGACTCGCCGGGCCCTATTCCCTGGAGATTCTGAGATTGACCAGCTCTTCCGGATCTTTCGGACTCTGGGGACCCCAGATGAGGTGGTGTGGCCAGGAGTTACTTCTATGCCTGATTACAAGCCAAGTTTCCCCAAGTGGGCCCGGCAAGATTTTAGTAAAGTTGTACCTCCCCTGGATGAAGATGGACGGAGCTTGTTATCGCAAATGCTGCACTACGACCCTAACAAGCGGATTTCGGCCAAGGCAGCCCTGGCTCACCCTTTCTTCCAGGATGTGACCAAGCCAGTACCCCATCTTCGACTCGGATTTCAGACGGTTCCCCGAATGCTGGCACTGTGGAGCAGACGCCCAAGAAGCCCGGCCTTCGACGCCAGACGTAAACAGCTCCGAATTAAGAATAATTCCTTGTTTATTAGATACATCACTGCTTTATGA-
Human
CDK2-
p27 Tail
Protein
Human
CDK2-
p27 Tail
MENFQKVEKIGEGTYGVVYKARNKLTGEVVALKKIRLDTETEGVPSTAIREISLLKELNHPNIVKLLDVIHTENKLYLVFEFLHQDLKKFMDASALTGIPLPLIKSYLFQLLQGLAFCHSHRVLHRDLKPQNLLINTEGAIKLADFGLARAFGVPVRTYTHEVVTLWYRAPEILLGCKYYSTAVDIWSLGCIFAEMVTRRALFPGDSEIDQLFRIFRTLGTPDEVVWPGVTSMPDYKPSFPKWARQDFSKVVPPLDEDGRSLLSQMLHYDPNKRISAKAALAHPFFQDVTKPVPHLRLGFQTVP
RMLALWSRRPRSPAFDARRKQLRIKNNSLFIRYITAL-
