# Supplementary figures and images for: Characterization of a naturally-occurring p27 mutation predisposing to multiple endocrine tumors
Source: Mol Cancer. 2010 May 21;9:116. doi: 10.1186/1476-4598-9-116 (PMC2881881; doi:10.1186/1476-4598-9-116)

## Slide 1
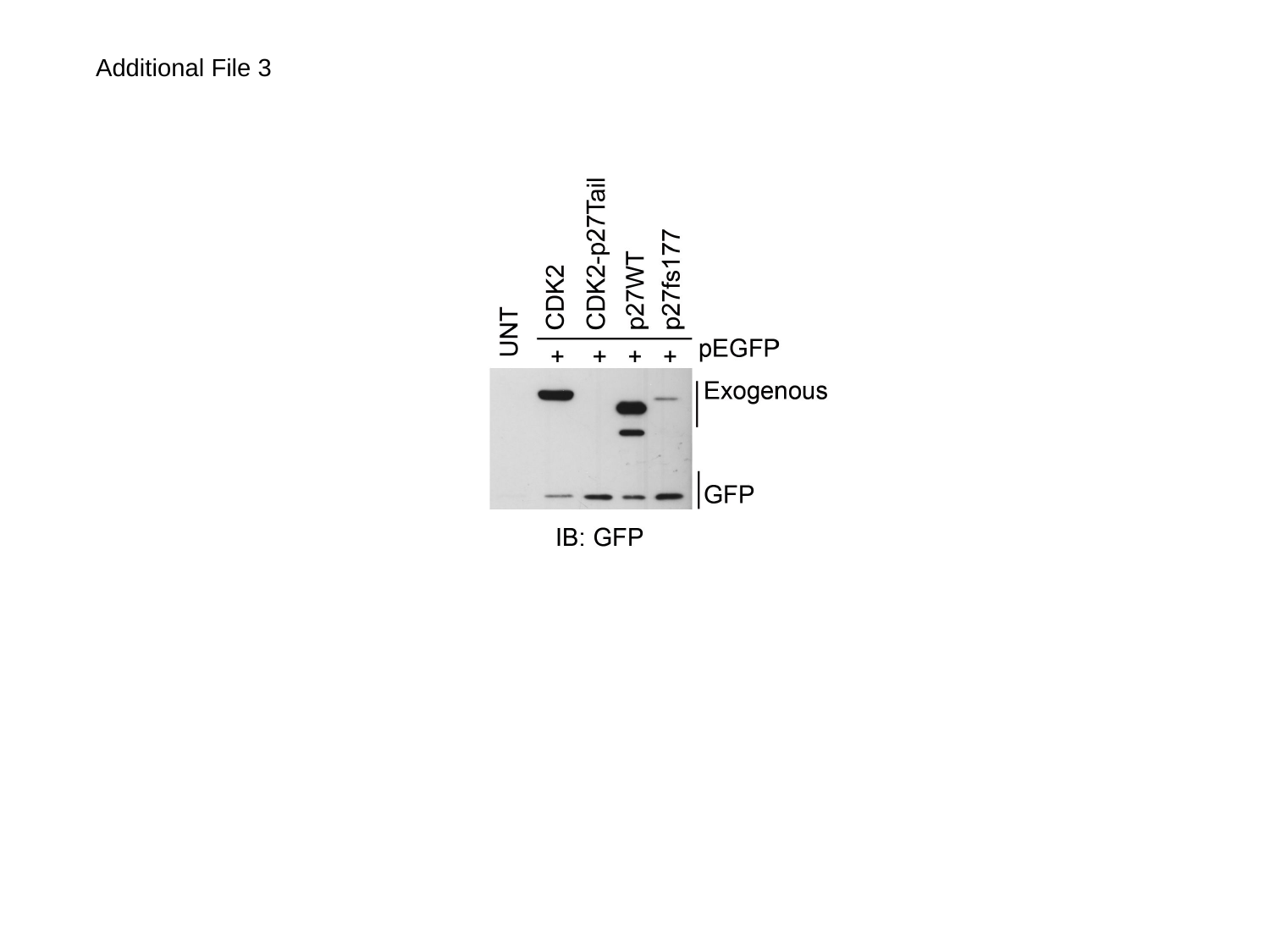

Additional File 3

Supplement: Additional file 3 — The low expression of CDK2-p27Tail is not due to lower transfection efficiency. MCF7 cells were co-transfected with the fusion constructs encoding GFP-CDK2, -CDK2-p27Tail, -p27WT or -p27fs177 and with an empty pEGFP vector to monitor the efficiency of transfection. Proteins were resolved and blotted with a monoclonal anti-GFP antibody. As mentioned in the article text, the CDK2-p27Tail protein is expressed at such a low level that it can be detected only following immunoprecipitation. [file 1476-4598-9-116-S3.PPT]

## Slide 1
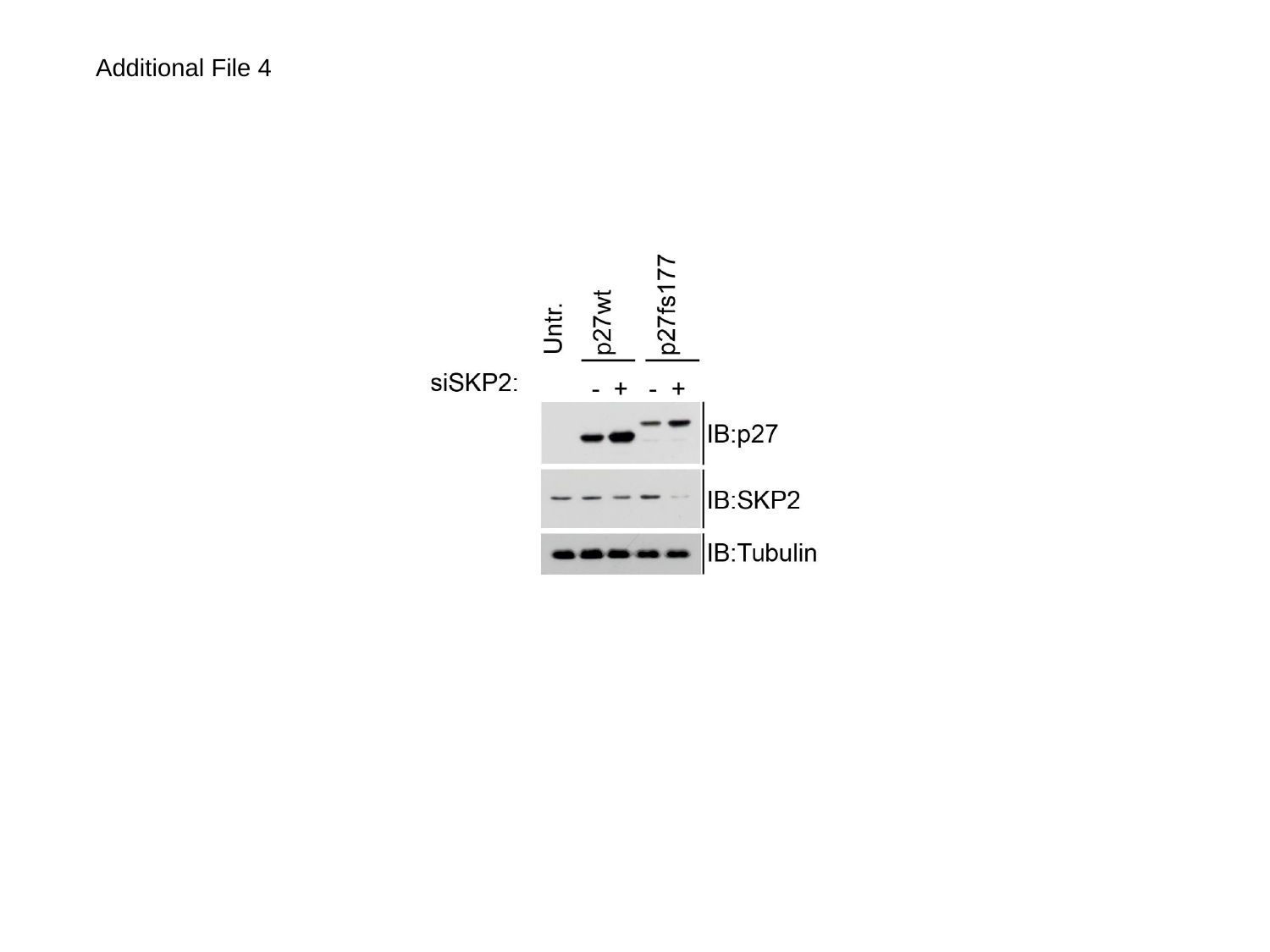

Additional File 4

Supplement: Additional file 4 — Degradation of p27fs177 occurs through Skp2-dependent pathways. For RNAi studies, short interfering RNA (siRNA) duplexes specific for Skp2 (n = 4) (1 mg) were transfected into MCF7 cells using X-tremeGENE reagent together with expression plasmids for p27wt (30 ng) or p27fs177 (50 ng). Immunoblotting was performed with the indicated antibodies against p27, SKP2 and α-Tubulin (to control for equal leading). [file 1476-4598-9-116-S4.PPT]

## Slide 1
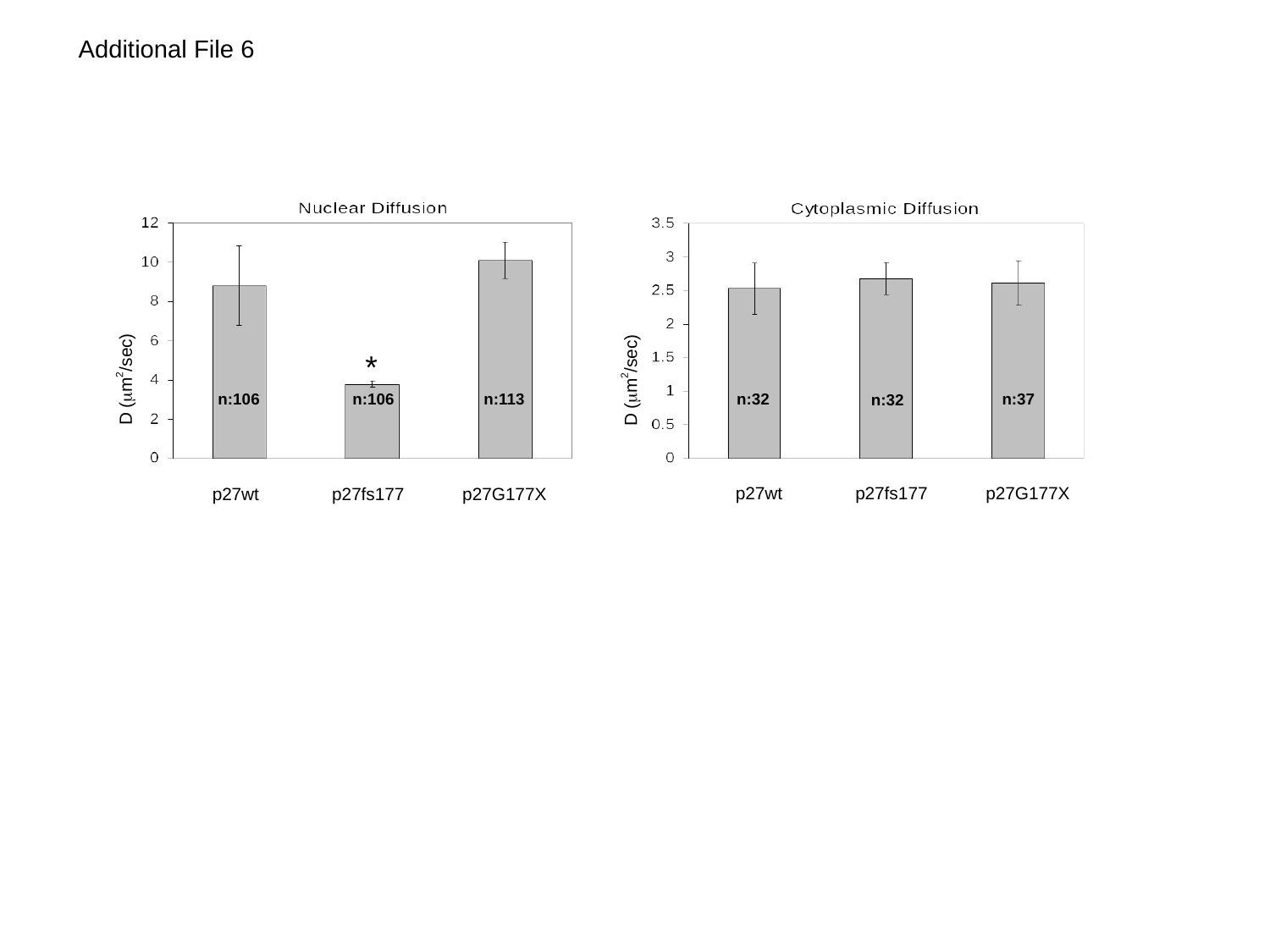

Additional File 6
*
D (μm2/sec)
D (μm2/sec)
n:106
n:106
n:113
n:32
n:37
n:32
p27wt p27fs177 p27G177X
p27wt p27fs177 p27G177X

Supplement: Additional file 6 — Photobleaching shows that p27fs177 has reduced motility in the nucleus, but not in the cytoplasm. Diffusion coefficient of the various GFP-p27 fusion proteins in the nucleus. The appropriate diffusion values are calculated as mean values of single measurements ± standard deviation. The number of bleached cells per construct is indicated in the diagram bar (n). The number of cells analyzed for cytoplasmic diffusion is smaller than for nuclear diffusione because only a percentage of cells (ca. 30%) shows cytopalsmic localization of these proteins. The asterisk (*) indicates a significant decrease of diffusion for thep27fs177 protein compared to p27wt (P = 0.0012). FRAP, fluorescence recovery after photobleaching. [file 1476-4598-9-116-S6.PPT]
