# Supplementary material for: Characterization of a naturally-occurring p27 mutation predisposing to multiple endocrine tumors
Source: Mol Cancer. 2010 May 21;9:116. doi: 10.1186/1476-4598-9-116 (PMC2881881; doi:10.1186/1476-4598-9-116)
Supplement: Additional file 5 — (a) p27fs177 is not phosphorylated at Ser10 in vitro. Hela cells were transfected with the p27wt, p27fs177 and p27S10A contructs. Cells were collected 24 h later. Proteins were resolved on 4-15% SDS-PAGE pre-cast gels and blotted with the antibodies indicated on the left side: polyclonal anti-P-S10 antibody and monoclonal antibodies against p27 and against α-tubulin. The arrow indicates the p27fs177 protein. For immunoprecipitation, 500 μg of protein lysates were incubated o.n. at 4°C with 10 ml of anti-P-S10 antibody and then immunoblotted with the monoclonal anti-p27 antibody. (b) p27fs177 interacts with Cdks. Stably-transfected p27fs177-Clone 9 cells were harvested while exponentially growing (pro) or after incubation for 72 hrs in medium supplemented with 0,1% FBS (serum starved, ss) and proteins immunoprecipitated o.n. at 4°C with 10 ml of anti-GFP polyclonal antibody, 20 ml of anti-CyclinD1 polyclonal antibody or 20 ml of anti-CyclinE polyclonal antibody. Proteins were resolved as in (a) and blotted with the antibodies indicated on the right side (Cdk2 and CyclinE monoclonal, Cdk4 and CyclinD1 polyclonal). [file 1476-4598-9-116-S5.PPT]

## Slide 1
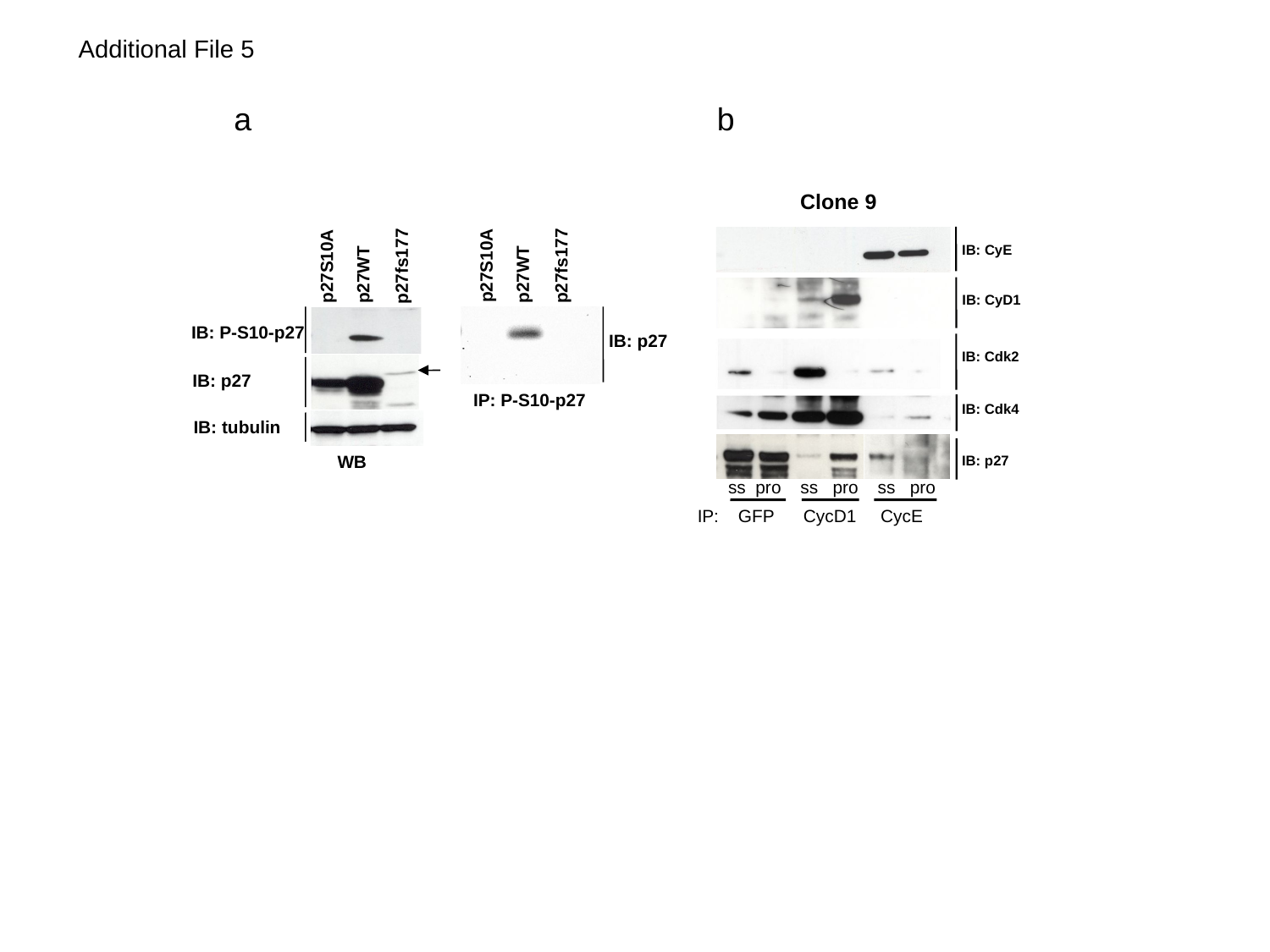

Additional File 5
a
b
Clone 9
IB: CyE
IB: CyD1
IB: Cdk2
IB: Cdk4
IB: p27
ss pro ss pro ss pro
IP: GFP CycD1 CycE
p27S10A
p27fs177
p27S10A
p27fs177
p27WT
p27WT
IB: P-S10-p27
IB: p27
IB: p27
IP: P-S10-p27
IB: tubulin
WB
