# Supplementary material for: Characterization of a naturally-occurring p27 mutation predisposing to multiple endocrine tumors
Source: Mol Cancer. 2010 May 21;9:116. doi: 10.1186/1476-4598-9-116 (PMC2881881; doi:10.1186/1476-4598-9-116)
Supplement: Additional file 7 — Expression of miRNA-221& 222 in rat adrenal tissues and in primary fibroblasts (REF cells) show no difference between normal and mutated rats. (a) Total RNA was extracted from normal rat adrenal tissue (wt/wt) and rat adrenal tumors (mut/mut) using Trizol (Invitrogen). Quantitation of mature miRNA-221 and 222 expression levels in and was performed by RT-PCR using TaqMan MicroRNA Assays. All RT-PCR were performed in triplicate. One endogenous control was used for the normalization of RNA input: small nucleolar RNA RNU44. The data are presented as the fold change of miRNA expression in tissues after normalization to an endogenous control (RNU44). (b) Exponentially growing REF7 (wt/wt) or REF10 (mut/mut) fibroblasts were collected. Total RNA extracted and TaqMan assays were performed as (a). The differences in expression between mutant and normal tissues or cells are not statistically significant. •Microsoft Power Point Presentation [file 1476-4598-9-116-S7.PPT]

## Slide 1
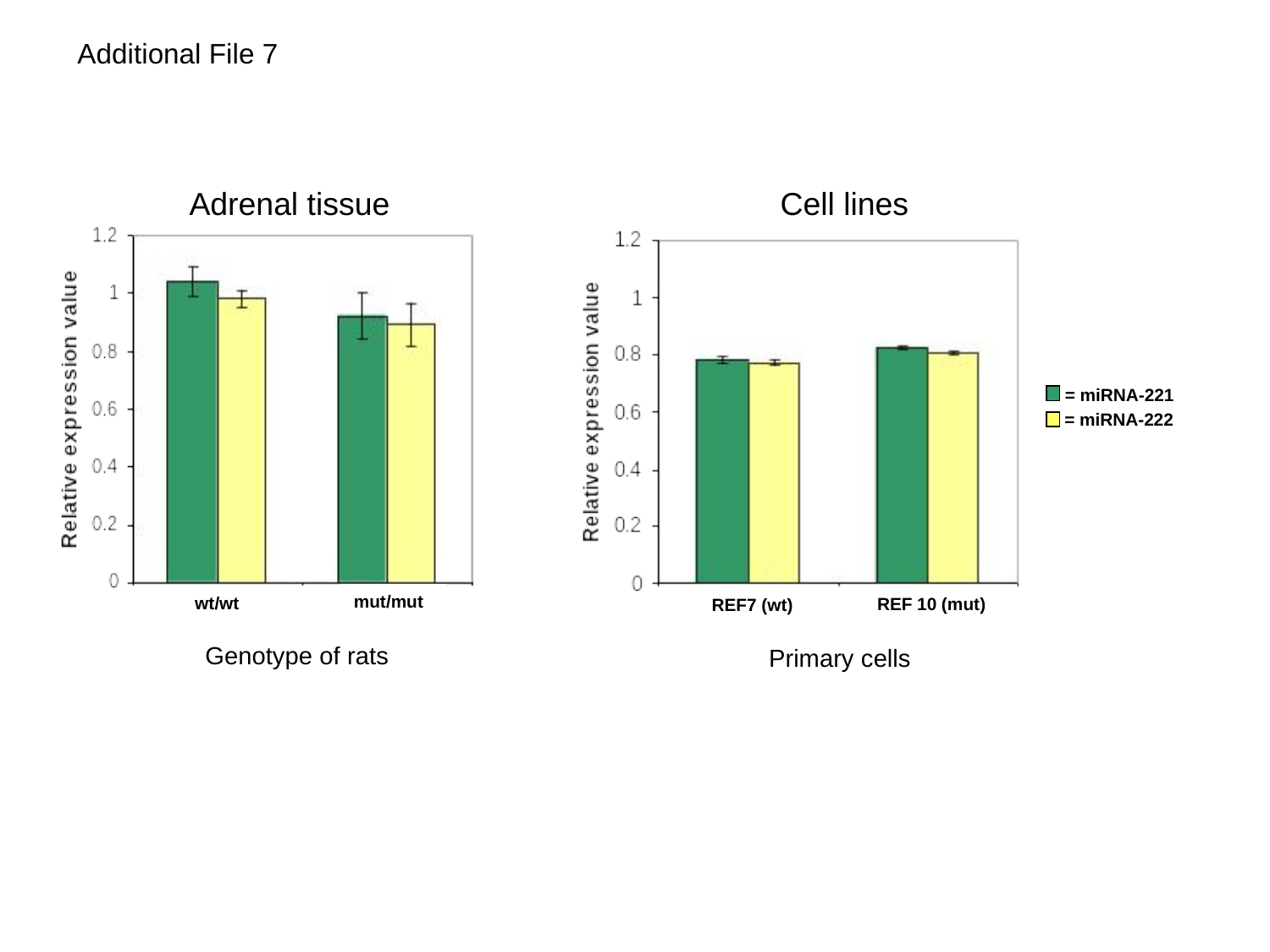

Additional File 7
REF 10 (mut)
REF7 (wt)
Primary cells
Adrenal tissue
Cell lines
mut/mut
wt/wt
Genotype of rats
= miRNA-221
= miRNA-222
