# Supplementary material for: Characterization of a naturally-occurring p27 mutation predisposing to multiple endocrine tumors
Source: Mol Cancer. 2010 May 21;9:116. doi: 10.1186/1476-4598-9-116 (PMC2881881; doi:10.1186/1476-4598-9-116)
Supplement: Additional file 8 — Supplementary Materials and Methods, Reference. Microsoft Word Document [file 1476-4598-9-116-S8.DOC]

**Supplementary Information**

**Supplementary Material and Methods**

*Indirect immunofluorescence staining.*

Cells grown on coverslips were fixed in 2% paraformaldehyde in PBS for 30 minutes at room temperature. Fixed cells were incubated 5 minutes in 0.25% TritonX in PBS, rinsed and blocked with 5% normal goat serum (Dako) in PBS for 30 minutes. Cells were incubated overnight at +4º C with the anti-p27 mouse monoclonal antibody (BD Biosciences) (1:200) in 5% normal goat serum, and then with a Cy3-conjugated secondary antibody (Zymed). Cell nuclei were stained with 1 μg/ml Hoechst for 5 minutes at room temperature and mounted on glass slides. One μM optical sections were generated using a Zeiss Axiovert 200 epifluorescence microscope including Apotome unit (Zeiss). Exposure time was 600 msec for wt p27-transfected cells and 1 sec for all other cells. Projections of 1-2 optical sections cutting across the nucleus were selected. Image processing was carried out with Zeiss computer software (AIM 3.2).

*Protein extraction*

For protein extraction from transfected cells, cells were collected, washed twice in PBS and lysed in lysis buffer for 20 min at 4°C. For regular western blotting, cells were resuspended in protein lysis buffer [10 mmol/l Tris-HCl (pH 7·4), 5 mmol/l EDTA, 130 mmol/l NaCl, 1% Triton, and 1X Mini-Complete protease inhibitors cocktail, Roche]. For immunoprecipitation, cells were resuspended in IP buffer (5mM EDTA, 0.5% Triton X-100 in PBS 1X). Lysates were spun at 12,000 *g* for 20 min and supernatants were collected. Protein concentration was assessed by the BCA assay method (Pierce Chemical Company). Total extracts were subjected to polyacrylamide gel electrophoresis using Bis-Tris 4-12% NuPAGE gels (Invitrogen) and the MES buffer (Invitrogen). Blotting was performed onto nitrocellulose membranes (Hybond-ECL, Amersham) and immunoblots were developed using West Pico chemoluminescent substrates (Pierce Chemical Company).

*Fluorescence recovery after photobleaching (FRAP).*

Rat 2 cells were spread on special Petri dishes containing a cover slip for microscopy at the bottom (MatTek Corporation; cells/dish). They were transfected the next day and FRAP measurements were started 24 hours after transfection. In order to get reproducible measurements, the circular bleaching area was set approximately in the middle of the nucleus. To mimic physiological conditions, a micro-incubation chamber (Zeiss) was installed on top of the objective table which allows investigation of the cells in an environment of 37 °C and 5% CO2 in air. For all experiments, a circular area of 9 µm2 was bleached with a 488 nm Argon laser. The laser power was adjusted to a maximum output (25 mW). For each measurement, four scans were taken prior to the bleaching and 116 scans to follow the recovery of fluorescence. Scanning times were 26.88 msec. All FRAP measurements and detections were carried out using a laser scanning microscope and software package provided by Zeiss (AIM Version 3.2). To verify the accuracy of our approach, the diffusion coefficient of the GFP protein alone was measured and the value (4.12 ± 1.07 µm2/sec) was in agreement with that reported in the literature (4.2 ± 1.2 μm2/sec) (1).

References

1. Yokoe H, Meyer T. (1996). Spatial dynamics of GFP-tagged proteins investigated by local fluorescence enhancement. *Nat Biotechnol* **14**:1252-1256.
